# Opening the Blood Brain Barrier with an Electropermanent Magnet System

**DOI:** 10.3390/pharmaceutics14071503

**Published:** 2022-07-20

**Authors:** Sahar Jafari, Ittai S. Baum, Oleg G. Udalov, Yichien Lee, Olga Rodriguez, Stanley T. Fricke, Maryam Jafari, Mostafa Amini, Roland Probst, Xinyao Tang, Cheng Chen, David J. Ariando, Anjana Hevaganinge, Lamar O. Mair, Christopher Albanese, Irving N. Weinberg

**Affiliations:** 1 Weinberg Medical Physics, Inc., North Bethesda, MD 20852, USA; sahar.jafari2011@gmail.com (S.J.); ittaisbaum@gmail.com (I.S.B.); udalovog@gmail.com (O.G.U.); xxt81@case.edu (X.T.); cxc717@case.edu (C.C.); anjana.heva@gmail.com (A.H.); lamar.mair@gmail.com (L.O.M.); 2 Department of Oncology, Georgetown University Medical Center, Washington, DC 20057, USA; yl285@georgetown.edu (Y.L.); rodriguo@georgetown.edu (O.R.); stanley.fricke@gmail.com (S.T.F.); albanese@georgetown.edu (C.A.); 3 Center for Translational Imaging, Georgetown University Medical Center, Washington, DC 20057, USA; 4 Department of Radiology, Georgetown University Medical Center, Washington, DC 20057, USA; 5 Independent Consultant, Oklahoma City, OK 73134, USA; jafary.maryam@gmail.com; 6 Department of Management Science and Information Systems, Oklahoma State University, Stillwater, OK 74078, USA; moamini@okstate.edu; 7 ACUITYnano LLC, Chevy Chase, MD 20815, USA; probstroland@gmail.com; 8 Department of Electrical and Computer Engineering, University of Florida, Gainesville, FL 32611, USA; dajo.ariando@gmail.com

**Keywords:** blood brain barrier, electropermanent magnet, magnetic resonance imaging

## Abstract

Opening the blood brain barrier (BBB) under imaging guidance may be useful for the treatment of many brain disorders. Rapidly applied magnetic fields have the potential to generate electric fields in brain tissue that, if properly timed, may enable safe and effective BBB opening. By tuning magnetic pulses generated by a novel electropermanent magnet (EPM) array, we demonstrate the opening of tight junctions in a BBB model culture in vitro, and show that induced monophasic electrical pulses are more effective than biphasic ones. We confirmed, with in vivo contrast-enhanced MRI, that the BBB can be opened with monophasic pulses. As electropermanent magnets have demonstrated efficacy at tuning B0 fields for magnetic resonance imaging studies, our results suggest the possibility of implementing an EPM-based hybrid theragnostic device that could both image the brain and enhance drug transport across the BBB in a single sitting.

## 1. Introduction

The blood brain barrier (BBB) is composed of brain endothelial cells, pericytes, and astrocytes in tight junctions without fenestrations [1,2,3,4]. The BBB prevents 98% of small molecules, and an even greater percentage of large molecules, from reaching their intended brain targets [5,6] and is therefore considered the most significant barrier to the efficient and targeted delivery of therapeutics to the brain [3]. Due to its significance, numerous approaches have focused on safely and temporarily opening the BBB. To date, heat [7,8,9], light [10,11,12], sound [13], electric fields [14], and magnetic fields [15] have all shown promise as BBB-opening techniques. Moving towards increased spatial specificity, image-guided BBB opening may be achieved using MRI-guided focused ultrasound (MRgFUS) [16]. Other techniques aim to bypass the BBB altogether by the delivery of therapeutic payloads to the spinal cord or via the nasal delivery of magnetic nanoparticles [5].

In cell culture models of the BBB, pulsed electric fields have been shown to be capable of destabilizing cell membranes and inducing the formation of nanoscale aqueous pores in the membranes, which may reseal (i.e., reversible electroporation) or may lead to cell death (irreversible electroporation) [6,14,16,17]. Drug infusion during reversible electroporation (using electric fields in the $\left(5-7\right)\times 10^{4}$ V/m range) has been used to improve survival and reduce tumor growth in glioma-bearing rats and dogs [18,19,20]. In a cell culture model, Sharabi et al. demonstrated that electric fields with significantly lower magnitudes (i.e., as low as 1500 V/m) could reversibly open the BBB [14]. These electric fields are similar in magnitude to those induced by clinical transcranial magnetic stimulation (TMS), as demonstrated by Hedarheydari et al. in mouse experiments involving components of a clinical TMS system [15].

Variations in electric field waveforms can have different effects on the brain. For example, Reilly et al. provided a theoretical basis that explained why monophasic pulses might be more effective at neuromodulation than the biphasic pulses typically used in TMS [21]. Peterchev et al. demonstrated a TMS power supply circuit that could apply currents with rapid rise times to TMS coils with slow fall times, effectively generating primarily monophasic electric pulses [22].

Electropermanent magnets (EPMs) confer users with the ability to specify material properties dynamically (i.e., to operate as programmable matter) [23]. EPMs generally consist of one or more permanent magnet material cores with a low magnetic coercivity (e.g., alnico), surrounded by a current-carrying coil [24,25]. By sending specified electrical currents through the coil, the permanent magnet component of the EPMs can be tuned to specific magnetizations [26]. Once tuned to a specific magnetization, the core material retains this magnetization without the need for the further application of current through the coil, and can be reset to a different magnetization with one or more additional current pulses. Appropriate control software allows these magnetizations to be programmed with a high spatial and temporal specificity. Udalov et al. modeled a control method using EPMs to generate electric fields in brain tissue, in which the magnetization status of the core would affect the rise- and fall-times of the magnetic field produced by the EPM (and hence also the waveform of the electric field induced by the magnetic field) [27]. Udalov et al. also presented models showing that the phases of current pulses in EPMs placed in arrays around the head could be adjusted such that superficial portions of the brain would be exposed to electric fields with different waveforms versus other parts of the brain. Such differential waveforms address regulatory limitations on scalp stimulation [28]. In earlier work on EPMs, Ropp et al. showed that EPMs could be used to perform nuclear magnetic resonance imaging [26]. In this work, we demonstrate the temporary opening of the BBB in both an in vitro cell culture model and in live mice using an array of four electropermanent magnets. We demonstrate variations in BBB opening for two different electric waveforms induced by the EPMs. This work represents the first use of EPMs with relevance to neuromodulation, BBB disruption, and drug delivery.

## 2. Materials and Methods

### 2.1. In Vitro Opening of a BBB Model System

In vitro experiments were performed on cell cultures containing tight junctions formed by Caco-2 cell monolayers (ReadyCell, Barcelona, Spain). Previous work has validated these Caco-2 cultures as BBB models [29,30]. Caco-2 cells were passaged in Eagle’s minimum essential medium (EMEM, ATCC, Manassas, VA, USA), then cultured on permeable transwell (TW) inserts in a 24-well plate at a density of 2 × 10^5^ cells/mL. Transwell dishes make use of inserts with track-etched membranes (Product #353096, Falcon, Corning Inc., Corning, NY, USA) for cell culture. After 21 days of culture in EMEM, Caco-2 cells form tight junction monolayers at the base surface of the insert in the culture well. We cultured and tested 18 TW samples possessing cell-loaded inserts with intact tight junction monolayers.

The cultures were exposed to electric fields induced by an array of four electropermanent magnets (described below). Prior to and after the electric field exposures, tight junction integrity was tested using transepithelial electrical resistance (TEER). TEER measurements were obtained with an EVOM^2^ epithelial volt/Ohm meter (World Precision Instruments, Sarasota, FL, USA).

**Figure 1:**
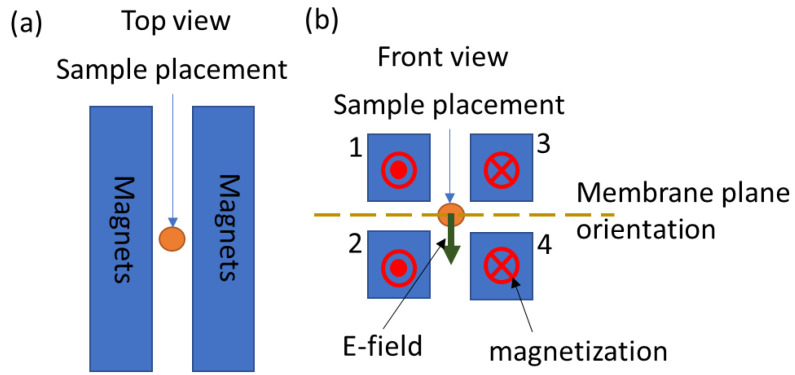
**Experimental geometry.** (**a**) Top view of the EPM array with a centrally positioned sample (orange circle). (**b**) Front view of the EPM array, showing four EPM assemblies with magnetizations directed along the length of each EPM assembly. The direction of the electric field is shown by an arrow, and a dotted line depicts the orientation of the transwell membrane (on which the Caco-2 cells are grown) for in vitro experiments. The EPM magnet assemblies are numbered 1 through 4.

Each EPM was composed of 200 Alnico-5 rods (each rod being 2 mm in diameter and 100 mm in length) bundled together into a rectangular bundle (measuring 3 cm × 3 cm × 20 cm). The EPM bundles were housed as assemblies in custom 3D-printed holders, around which were wound 40 turns of magnet wire (14 HML NEMA MW16-C, MWS Wire Industries, Oxnard, CA, USA) for applying current pulses. An array of four EPM assemblies is shown in Figure 1. The distance between the magnets is of the order of 1 cm. The sample is placed in the center between the magnets. Thus, the distance between the magnets and the samples is also of the order of 1 cm.

**Figure 2:**
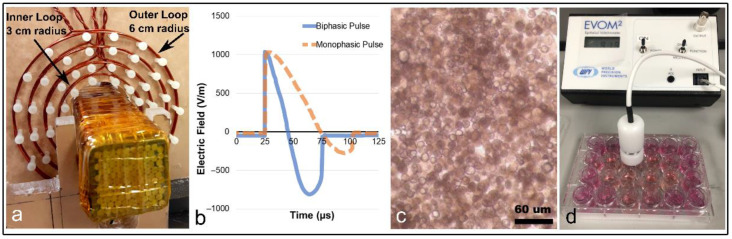
**EPM assembly electric field measurements, Caco-2 cultures, and TEER measurement apparatus.** (**a**) Electropermanent magnet assembly and coated copper wire windings, centered in an electric-field measurement device composed of concentric coils. The electric field generated by pulsing the EPM assembly is quantified by measuring the voltage induced across the coils. (**b**) The induced electric field for biphasic and monophasic pulses. As can be seen in (**b**), the monophasic pulses (orange dotted line) induce only a one-directional electric field, while the biphasic pulses (solid blue line) induce bi-directional electric fields. (**c**) Caco-2 cells cultured on transwell membranes prior to TEER measurements. (**d**) Apparatus and technique for TEER measurements of the BBB tight junction model.

Pulsing the coils of the EPM assemblies with 1 kiloampere currents was achieved using an H-bridge circuit. The EPM array was able to generate magnetic fields of up to 150 mT at the sample position. The duration of the current pulse was about 50 μs as shown in Figure 2. Experiments with a single EPM assembly were performed to measure the electric field produced by a single EPM assembly (Figure 2a,b). These experiments showed that the single EPM assembly produced an electric field in the range of 1000 V/m at 1 cm distance from the EPM assembly’s surface.

In the four EPM assembly array (Figure 1), the direction of the electric field produced in the sample during the pulse can vary depending on the magnetization direction of the magnets and on the sign of the electric current pulses. For the experiments reported here, the electric field was oriented perpendicularly to the cell culture membrane plane.

Depending on the initial magnetization states of the EPM assemblies, two different kinds of electric field pulses can be produced. If the magnets are saturated and the current pulses apply a magnetic field in the direction of the magnets’ magnetization, then the EPM assembly’s magnetization state before and after the current pulse will not change. In this case, the electric field pulse will be a biphasic (BP) pulse, where both positive and negative electric fields are generated during the pulse and the time integral of the electric field in the sample is zero. If the EPM assemblies were initially saturated, but the coil field during the pulse is opposite to this initial magnetization state, then the EPM assemblies will become demagnetized during the pulse. In this case, the electric field pulse is a mono-phasic (MP) pulse, meaning that the time integral of the electric field is non-zero, and the sample is primarily exposed to an electric field with a single polarity.

A single EPM assembly and corresponding electric field measuring setup are shown in Figure 2a. Coils of varying diameter were set up to measure the induced currents, with the smallest diameter being 3 cm and the largest diameter 6 cm. Measurements of the electric field for both MP and BP pulses are shown in Figure 2b. As shown in Figure 2b, the electric field produced by the BP pulse lasted 50 μs, with approximately equal positive and negative electrical lobe magnitudes and durations. The MP pulses were below 75 μs, but primarily produced a unipolar electric field.

We separated the 18 Caco-2 TW cultures into three groups of equal size (*n* = 6 TW cultures per group): control (no magnetic field exposure), biphasic pulse (BP) electric field exposure, and monophasic pulse (MP) electric field exposure. For all three groups, the tight junctions of the cells were assessed using the EVOM^2^ to measure the TEER [31]. We used an STX2-PLUS electrode (World Precision Instruments), calibrated using a 1000 Ω probe. The STX2-PLUS electrodes were treated with chloride (4.5% sodium hypochlorite, CLOROX, California USA) for >10 min prior to TEER measurements. Before and after each experiment, the probe was rinsed with isopropanol and deionized water, then dried in air.

For groups 2 and 3, a series of 40 pulses (BP or MP) was applied over 5 min. Then, the TEER measurements were repeated. The controls, biphasic pulse exposure, and monophasic pulse exposure samples were each tested six times (*n* = 6).

### 2.2. In Vivo Experiments on Mice

In vivo experiments were performed in the Preclinical Imaging Research Laboratory at Georgetown University Medical Center, with all experiments being performed in accordance with the USA standards for animal well-being set by the Office of Laboratory Animal Welfare, and in accordance with the Georgetown University Medical Center Institutional Animal Care and Use Committee (IACUC, protocol #2019-0048).

**Figure 3:**
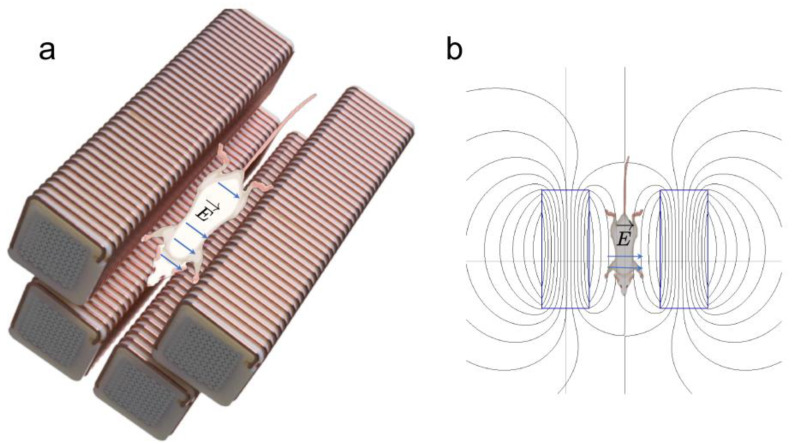
**Murine experimental setup.** (**a**) For in vivo experiments, a mouse was placed inside the four-magnet EPM array, with the length of the mouse oriented along the length of the EPM assemblies. (**b**) The EPM assemblies, shown as blue rectangles, generate magnetic fields, shown here as black lobe-shaped lines. The magnetic pulses generate electric fields (blue arrows) to which the mouse is exposed.

A total of 16 healthy C57Bl/6 male mice (~1 year of age, weighing between 28 and 30 g) were divided into two groups (sham and treated). The mice in the treatment group were anesthetized with isoflurane, injected intra-peritoneally with the gadolinium-based contrast agent Gadovist (BayerHealthCare AG, Leverkusen, Germany) at a dose of 0.1 mmol/kg and exposed to 40 MP pulses (as described above in the in vitro studies section) at a comparable distance from the mouse’s brain (Figure 3). The mice in the sham group received only the contrast agent injection. Twenty minutes after injection, all animals underwent magnetic resonance imaging (MRI) in the Georgetown-Lombardi Preclinical Imaging Research Laboratory on a 7 tesla Bruker Biospec Avance NEO magnet run by ParaVision 360 software. Briefly, the mice were anesthetized (1.5% isoflurane in a gas mixture of 30% oxygen and 68.5% nitrous oxide) and placed on a custom-manufactured stereotaxic device with built-in temperature and cardio-respiratory monitoring (ASI Instruments, Warren, Michigan). The MRI protocol used was a T1-weighted RARE sequence with TE: 8.75 ms, TR: 850 ms, rare factor: 2, matrix: 256 × 256, and FOV: 20 × 20 mm. BBB permeability was evaluated by measuring T1 signal intensity enhancement in two regions of interest (ROI): one was localized on the right brain cortex, the other on the right neck muscles (which served as a positive control for contrast agent uptake) and background air (which was used to normalize for image intensity). Four of the treated mice were randomly selected for follow-up studies to assess the duration of BBB opening. These mice were re-injected with the contrast agent and re-imaged by MRI again 24 h after their initial testing.

### 2.3. Statistical Analysis

The TEER data were evaluated for normality through Shapiro–Wilk and Anderson–Darling tests. One-way analysis of variance (ANOVA) and Fisher’s least significant difference (LSD) tests were performed for statistical investigation between the three groups. A statistical analysis of the MRI signal intensity over the two regions of interest (ROIs) was performed using JMP statistical software V16.1 (SAS, Cary, NC, USA). Shapiro–Wilk and Anderson–Darling tests were applied to test the normality of the distribution. JMP provides the Shapiro–Wilk test in the distribution platform for a departure from a normal distribution within each group of experiments. This test has been shown to be more powerful than alternative tests, including the Anderson–Darling test [32]. ANOVAs followed by Fisher’s LSD (where we had more than two experimental groups) were used to compare the efficiency of different pulsing regimes. Here, *p* < 0.05 was considered significant in the rejection of the null hypothesis. Fisher’s LSD method is more commonly used to compare means from multiple processes, as it compares all pairs of means and controls for error rate for each individual pairwise comparison but does not control the family error rate. Both error rates are given in the output. A t-test analysis was used to compare the ROI measurement of MRI signal intensities between the control and test groups of mice. Here, *p* < 0.05 was considered significant in the rejection of the null hypothesis that there was a difference between the control and test groups. Due to the small sample size (four mice) for the 24-h delayed mouse studies, observational assessment only was performed for this experiment.

## 3. Results

### 3.1. Pulse Characterization and In Vitro Model BBB Culture Experiments

**Figure 4:**
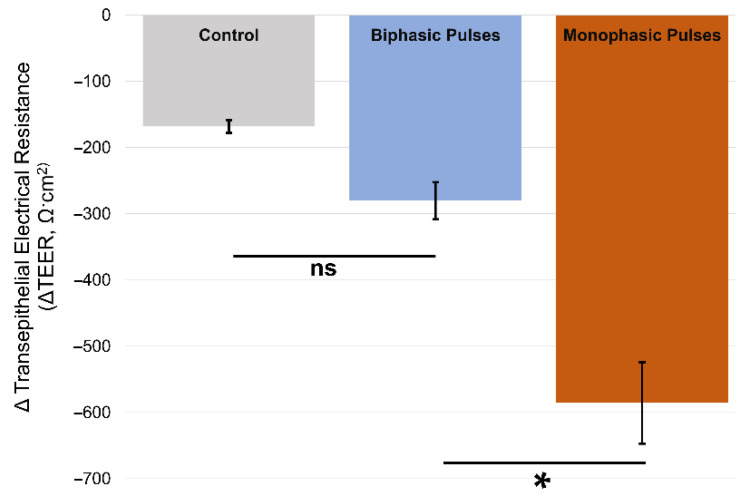
**TEER measurement results.** Samples exposed to monophasic electric field pulses demonstrate statistically relevant changes in TEER measurements. Here, ns indicates results which are not statistically significant, while * indicates statistical significance.

Initial measurements of resistance in all 18 cultures confirmed the existence of tight junctions across the transwell membranes, indicating that the cultures were intact and had formed sufficiently conformal layers with accompanying tight junction connections, as in prior work describing this in vitro BBB model [33,34,35,36]. All of the initial resistance measurements reported resistances of approximately 2000 Ω·cm^2^ or greater, confirming the formation of tight junctions. The TEER measurements of the control group (no magnetic field) showed a change of less than 200 Ω·cm^2^ in resistance over the course of the experimental timeframe. Samples exposed to biphasic pulses exhibited non-significant changes in TEER values versus controls (*p* = 0.4), while samples exposed to MP pulses experienced the highest change in TEER resistance as compared with controls (*p* < 0.01) (Figure 4).

### 3.2. In Vivo BBB Opening Experiments

**Figure 5:**
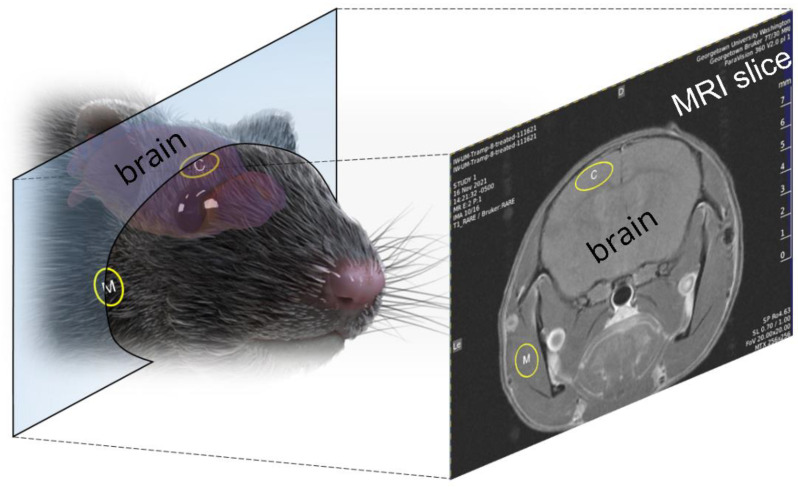
**Mouse brain MRI for assessing Gadovist uptake.** MRI slices collected of the mouse head were analyzed at two distinct locations, shown above (yellow circles). Gadovist uptake, as characterized by a change in signal intensity before and after EPM pulses, was measured in regions of the cortex (yellow circle with (**C**)) and regions of muscle (yellow circle with (**M**)) for comparison.

**Figure 6:**
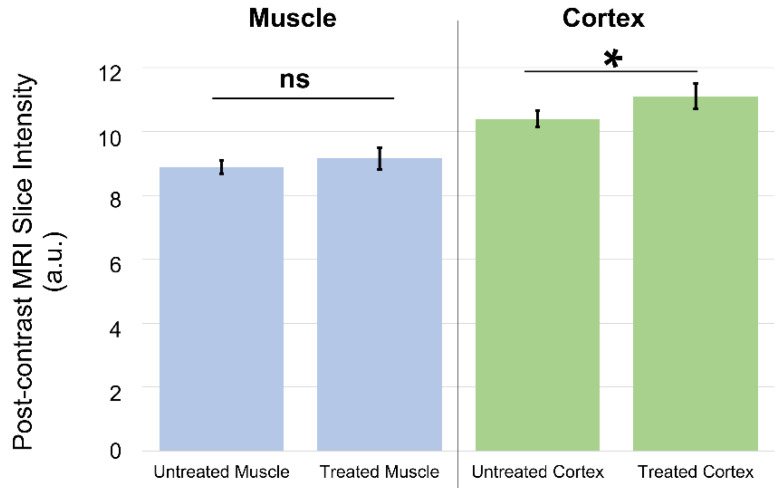
**In vivo Gadovist uptake results for muscle and cortex.** Comparison of MRI region-of-interest signal in muscle and brain cortex for untreated and treated mice. The muscle shows no statistically significant difference in post-contrast MRI intensity for untreated and treated animals. The cortex shows a statistically significant difference in post-contrast MRI intensity for untreated animals as compared with treated animals. Here, ns indicates results which are not statistically significant, while * indicates statistical significance.

As only MP pulses were used in vivo, we simply refer to groups as sham or treated. Figure 5 depicts MRI slices taken from mice, showing locations for comparing Gadovist uptake in the brain cortex versus Gadovist uptake in the muscle. Figure 6 shows data on the averaged Gadovist uptake in the brain cortex and facial muscle, comparing the control group (sham) with the treated group (MP pulses). Data, in the form of the average MR image intensity across a circular region of a single slice for each mouse (Figure 5), at specified locations (muscle, cortex), were first evaluated for normality through Shapiro–Wilk and Anderson–Darling tests. The *p*-values obtained from the Shapiro–Wilk tests were 0.49 and 0.16 for the brain-cortex-to-muscle ratio for the control and treated groups, respectively. The *p*-values from the Anderson–Darling normality tests for the cortex-to-muscle ratio were 0.51 and 0.23 for the control and treated groups, respectively. The *p*-values for the brain cortex of the control and treated groups from the Shapiro–Wilk tests were 0.6 and 0.5, respectively. The *p*-values from the Anderson–Darling normality tests for the brain cortex were 0.6 and 0.5 for the control and treated groups, respectively. The *p*-values from the Shapiro–Wilk tests for the muscle of the control and treated groups were 0.6 and 0.08, respectively. The *p*-values from the Anderson–Darling normality tests for the muscle control and treated groups were 0.6 and 0.1, respectively. Here, *p*-values > 0.05 indicated that the normal distribution was a good fit for each group. A t-test analysis was performed for statistical investigation between the groups of experiments. A *p*-value of less than 0.05 (typically < 0.05) was considered statistically significant.

There was a significant difference between the contrast agent uptake of the brain cortex (*p* < 0.0002) in the animals of the treated group and the control group. There was a significant difference (*p* < 0.0003) between the cortex-to-muscle ratio in the animals of the treated group and the control group as well. No significant difference (*p* < 0.2) was observed in the contrast agent uptake by the muscles of the treated group and the control group. The number of animals kept for 24 h was too small to make statistically significant observations. We infer that the BBB stays open for a time greater than the initial scan (20 min), but less than 24 h.

**Figure 7:**
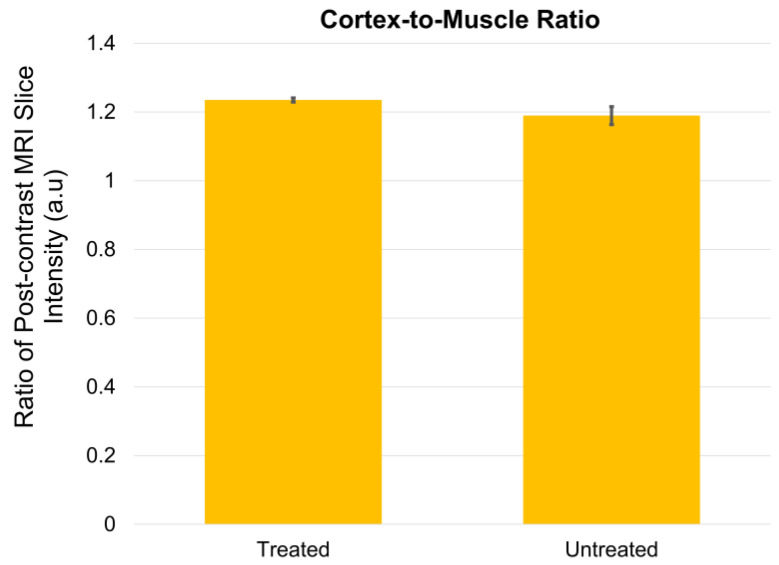
**Ratio of cortex-to-muscle post-contrast MRI intensity in mice.** Mice treated with MP pulses showed a greater ratio of cortex-to-muscle post-contrast intensity as compared with untreated mice.

Comparing ratios for the treated and untreated animals, a cortex-to-muscle ratio was calculated for the treated and untreated groups (Figure 7). The ratio simply takes the post-contrast MRI slice intensities for the cortex and divides by the post-contrast slice intensities for the muscle, separately for treated and untreated animals.

## 4. Discussion

Our in vitro results demonstrate that a monophasic electric field is more than twice as effective at opening tight junctions in a BBB model as compared with a biphasic electric field, consistent with prior theoretical results for TMS efficacy [21]. Consistent with prior work [6,14,15,18], the increased MRI signal in the cortex of treated mice likely derives from the disruption of the BBB (and the release of Gadovist from the local vasculature, see right side of Figure 6) by rapid magnetic pulses generated by our electropermanent magnet system. No significant increase in MRI signal was noted for non-brain tissues (i.e., muscle, see left side of Figure 6). The results of the murine in vivo experiments demonstrate that a monophasic electric field produced by EPMs is effective in temporarily opening the BBB. It should be noted that the MP pulse is not strictly monophasic, but does include a short ~25 µs negative voltage component (Figure 2b). This negative voltage component of the MP may or may not be implicated in the BBB opening results observed. Future experiments will determine if the small negative component of the MP pulse is significant or not. As the phase control of the EPM-generated magnetic fields can create different waveforms (i.e., monophasic versus biphasic) at different locations in the brain, and can also be used to create magnetic resonance images, these results demonstrate that EPMs hold promise as novel devices for enhancing drug delivery via localized BBB opening [27]. Since EPM arrays have previously demonstrated use in novel methods of field-tunable NMR and MRI [26], we envision systems built with such arrays used as tools for combined imaging and therapy. A popular technique for disrupting the BBB is focused ultrasound, which may have the disadvantage of causing permanent neuronal damage and therefore has a relatively low therapeutic ratio [37,38]. Based on literature showing that the electrical field required for permanent neuronal damage is at least 100 times higher than the fields we are realizing with electropermanent magnets [39], we expect a more favorable therapeutic ratio. An additional potential advantage is the use of the same electropermanent magnet array to collect MRI images with the same apparatus, which will be convenient for clinicians [27]. Finally, the data demonstrate that BBB disruption is dependent on the waveform of the electrical pulses generated by the electropermanent magnets. We expect that this principle can be taken advantage of by selectively actuating the electropermanent magnets with appropriate phases, thereby implementing spatially selective BBB opening over preselected tracts and regions of the brain with arbitrary shapes and sizes.

## 5. Conclusions

Our results demonstrate that the BBB may be opened selectively and temporarily through an array of electropermanent magnets that can be dynamically controlled to yield monophasic or biphasic electric fields. The findings are promising as a proof-of-principle for the construction of a hybrid EPM-based device with multiple functionalities, including MRI, TMS, and drug delivery. The ability to tailor electrical waveforms, and hence selectively affect BBB permeability according to the location in the brain, would be useful in focal drug delivery in the brain (enabling a reduction the off-target effects of such drugs). Additional studies will need to be conducted to establish the limits of such a system in terms of drug size and localization efficiency.

## Data Availability

Data is contained within the article.
